# Autoimmune lymphoproliferative syndrome: more than a FAScinating disease

**DOI:** 10.12688/f1000research.11545.1

**Published:** 2017-11-01

**Authors:** Karen Bride, David Teachey

**Affiliations:** 1 Division of Oncology, The Children’s Hospital of Philadelphia, Philadelphia, PA, USA

**Keywords:** autoimmune, cytopenias, sirolimus, MMF, double negative T cells, lymphoproliferative disease, Fas/FasL, Targeted therapy

## Abstract

Autoimmune lymphoproliferative syndrome (ALPS) is an inherited syndrome characterized by abnormal lymphocyte survival caused by failure of apoptotic mechanisms to maintain lymphocyte homeostasis. This failure leads to the clinical manifestations of non-infectious and non-malignant lymphadenopathy, splenomegaly, and autoimmune pathology, most commonly, autoimmune cytopenias. Since ALPS was first characterized in the early 1990s, insights in disease biology have improved both diagnosis and management of this syndrome. Sirolimus is the best-studied and most effective corticosteroid-sparing therapy for ALPS and should be considered first-line for patients in need of chronic treatment. This review highlights practical clinical considerations for the diagnosis and management of ALPS. Further studies could reveal new proteins and regulatory pathways that are critical for lymphocyte activation and apoptosis.

## Background

Autoimmune lymphoproliferative syndrome (ALPS) is a rare condition characterized by defective apoptotic mechanisms that disrupt lymphocyte homeostasis ^1– 4^. Apoptotic defects lead to a lymphoproliferative disease with clinical manifestations, including lymphadenopathy, hepatomegaly, splenomegaly, autoimmune disease, and secondary malignancies. Characterized initially in the 1990s, ALPS was noted in a cohort of patients with chronic lymphoproliferation and an increased number of a characteristic T-cell population termed “double negative T cells” (DNTs) ^5^. DNTs express CD3 and the alpha/beta T-cell receptor (TCRα/β) but lack CD4 and CD8. Other signature laboratory abnormalities in ALPS include elevated levels of interleukin 10 (IL-10), IL-18, vitamin B _12_, soluble FAS ligand (sFASL), and IgG in plasma or sera as well as i *n vitro* evidence of defective FAS-mediated apoptosis ** ^3, 4, 6, 7^. Significant advances in our understanding of the pathophysiology of ALPS led to improved diagnostic criteria and eventually targeted therapeutic strategies, including the mammalian target of rapamycin (mTOR) inhibitor sirolimus (also known as rapamycin or Rapamune). Though considered a rare disease, ALPS is now more commonly diagnosed, as more clinicians have become aware of the disorder. Over the past 10–15 years, improvements in genomic technologies have led to the description of a number of ALPS-like autoimmune and lymphoproliferative disorders, including *RAS*-associated leukoproliferative disease (RALD); caspase-8 deficiency state (CEDS); *p110delta* activating mutation causing senescent T cells, lymphadenopathy, and immunodeficiency (PASLI or activated PI3K delta syndrome); *CTLA-4* haploinsufficiency with autoimmune infiltration (CHAI); gain-of-function (GOF) signal transducer and activator of transcription 3 ( *STAT3*) mutations; and lipopolysaccharide-responsive vesicle trafficking, beach and anchor containing ( *LRBA*) deficiency with autoantibodies, regulatory T-cell defects, autoimmune infiltration, and enteropathy (LATAIE) ( Table 1) ^8, 9^. Collectively, these rare conditions clinically resemble ALPS and often are misdiagnosed as ALPS. Management for these conditions may include targeted therapy or hematopoietic stem cell transplant (HSCT) or both; therefore, it is crucial that clinicians understand and recognize how to diagnose and manage ALPS and these ALPS-like disorders.

**Table 1.** Autoimmune lymphoproliferative syndrome (ALPS)-related syndromes that are potentially similar to but genetically distinct from ALPS or meet characteristics of ALPS with undetermined genetic defects (ALPS-U).

ALPS-related syndromes					
Disease	Nomenclature	Mutation	Clinical features	Laboratory biomarkers	Potential targeted therapies
Ras-associated autoimmune leukoproliferative disorder ^65^	RALD	Germline or somatic *NRAS* and *KRAS* mutations RAS markedly decreases Bim protein expression leading to impaired lymphoid withdrawal and T-cell receptor (TCR)- induced apoptosis.	Primary immunodeficiency disorder of defective apoptosis leading to lymphadenopathy, massive splenomegaly, increased circulating B cells, hypergammaglobulinemia, and autoimmunity increased risk for hematopoietic malignancies.	Persistent absolute or relative monocytosis, hypergammaglobulinemia, B lymphocytosis Does not exhibit elevated “double negative T cells” (DNTs), vitamin B _12_ Activating somatic mutations in *KRAS* or *NRAS*	Mitogen-activated pathway kinase (MAPK) inhibitors (for example, trametinib), mammalian target of rapamycin (mTOR) inhibitors (sirolimus, everolimus)
Dianzani autoimmune lymphoproliferative disease ^32, 66^	DALD	No causative genes identified Overexpression of the cytokine osteopontin ^67^ Perforin ^68^	Exhibit autoimmunity, lymphoproliferation, splenomegaly, and defective Fas without expansion of DNT cells	Absent DNTs FAS resistance but without *FAS* or *FASL* mutations	
Caspase-8 deficiency state ^38^	CEDS	Loss-of-function mutation in *CASP8* thought to play a dual role in the induction of the nuclear factor-kappa B (NF-κB) transcription factor during lymphocyte activation as well as in apoptosis mediated by the Fas death-inducing signaling complex (DISC)	Exhibit lymphoproliferation and apoptosis defects observed in ALPS but manifests immunodeficiency rather than autoimmunity; recurrent sinopulmonary infections ^69^. Increased risk for malignancy	Serum Ig levels, antibody function, lymphocyte activation Defective activation of T, B and natural killer (NK) cells *CASP8* deficiency	
Fas-associated death domain deficiency ^70^	*FADD* deficiency	Autosomal recessive (AR) *FADD* deficiency	Characterized by severe bacterial and viral infections, congenital heart defects and recurrent episodes of fever, liver dysfunction, and seizures	*FADD* deficiency	
Common variable immunodeficiency 9 ^71^	Protein kinase C delta ( *PRKCD*) deficiency	AR *PRKCD* primary immunodeficiency	Characterized by recurrent infections, lymphadenopathy, hepatosplenomegaly, autoimmunity, and NK cell dysfunction	IL-10 overexpression by B cells	
Activated PI3K delta syndrome ^29, 48, 72^	APDS, also known as PASLI	Heterozygous gain-of-function mutations in *PI _3_KCD* or *PI _3_KR1*	Recurrent respiratory infections and increased susceptibility to viral infections with both B- and T-cell defects	Decreased naïve T cells, low IgG, IgA, and normal or elevated IgM	mTOR inhibitors, PI3K inhibitors
X-linked immunodeficiency with magnesium defect, Epstein-Barr virus (EBV) infection and neoplasia ^28^	XMEN disease	Loss-function mutations in magnesium transporter 1 ( *MAGT1*); X-linked	Chronic high-level EBV with increased EBV-infected B cells and increased susceptibility to EBV-associated lymphomas	Mg deficiency	Magnesium
Gain-of-function mutations in signal transducer and activator of transcription 1 defect	GOF *STAT1* defect	*STAT1-*gain of function mutation	Chronic mucocutaneous candidiasis, recurrent *Staphylococcus aureus* infections, cerebral aneurysms, and multiple autoimmune features	Decreased TH17 response	JAK/STAT inhibitors (for example, Ruxolitinib)
Gain-of-function mutations in signal transducer and activator of transcription 3 ^31, 48^	GOF *STAT3*- mutations	*STAT3*-gain of function mutation	Lymphoproliferation and childhood-onset autoimmunity thought to result from dysregulated cytokine signaling and interstitial lung disease		Anti-IL-6R monoclonal antibody (Tocilizumab)
Cytotoxic T lymphocyte antigen ( *CTLA4*) haploinsufficiency with autoimmune infiltration ^8, 9^	CHAI	Heterozygous loss-of-function mutations in *CTLA4*	Hypogammaglobulinemia and autoantibody-mediated cytopenias, lymphadenopathy, splenomegaly, organ- specific autoimmunity, and lymphocytic infiltration of non-lymphoid organs CHAI more commonly seen in older children or young adults while disease onset in LATAIE is typically earlier.		CTLA4-Ig fusion drug (Abatacept) mTOR inhibitors
Common variable immune deficiency caused by defect in lipopolysaccharide- responsive and beige- like anchor protein ^8, 9, 48^ LRBA deficiency with autoantibodies, regulatory T-cell defects, autoimmune infiltration, and enteropathy	*LRBA* deficiency LATAIE	*LRBA* encodes the lipopolysaccharide-responsive and beige-like anchor protein, thought to regulate CTLA4 (cytotoxic T lymphocyte antigen-4)	Antibody deficiency, infection, autoimmunity, and lymphoproliferation, often linked with enteropathy or inflammatory bowel disease. Lymphocyte infiltration also seen in lungs and brain		CTLA4-Ig fusion drugs Hydroxycholoroquine or chloroquine mTOR inhibitors

The majority of these syndromes have been defined based on the genomic defect with associated symptoms. GOF, gain-of-function.

## Pathophysiology

**Figure 1.**
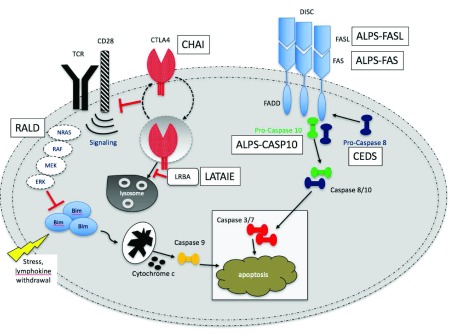
Fas apoptotic pathway. In an attempt to downregulate an immune response, activated B and T cells upregulate FAS while activated T cells will activate FAS ligand (FASL). These cells will interact and trigger a caspase cascade, leading to proteolysis, DNA degradation, and apoptosis. This FAS-mediated pathway is part of the extrinsic apoptotic pathway. In contrast, mitochondrial-induced apoptosis after cellular stress is part of the intrinsic apoptotic pathway.

Normal control of lymphocyte proliferation and immune tolerance is essential for both host defense and protection against self-directed immune attack ^10, 11^. This process is most often mediated by the cell surface receptor FAS, a member of the tumor necrosis factor receptor (TNFR) superfamily, also termed CD95/APO1 ( Figure 1) ^10^. Similar to others in the TNFR family, FAS operates as a homotrimeric complex and is activated by the cognate FAS ligand (FASL), another homotrimeric protein complex homologous to TNF ^12^. Following ligation, the intracellular death domain of FAS nucleates an extended complex of the Fas-associated death domain (FADD) adaptor protein with caspase-8 and -10. These caspases subsequently undergo a signaling cascade of downstream effector caspases and other targets that eventually lead to proteolysis, DNA degradation, and apoptosis. Mutations in genes encoding even a single defective subunit in FAS, FASL, FADD, and CASP10 have all been linked to ALPS and have been classified according to their genetic defect ( Table 2).

**Table 2.** Prior classification of genetic mutations related to autoimmune lymphoproliferative syndrome, according to the underlying genetic defect.

Type	Disease	Mutation	Proportion of ALPS attributed to mutation of the gene
Type 0	ALPS-FAS	Germline homozygous mutations in *FAS*	
Type Ia	ALPS-FAS	Germ-line heterozygous mutations in *FAS*	65–70%
Type Im	ALPS sFAS	Somatic mutation in *FAS*	15–20%
Type Ib	ALPS-FASLG	Germline mutations in FASL ( *TNFSF6*)	<1%
Type IIa	ALPS caspase 10	Germline mutation in *CASP10*	3–6%
Type III	ALPS-U	No identifiable mutation	20%

Prior classification of genetic mutations related to autoimmune lymphoproliferative syndrome, according to the underlying genetic defect ^6, 73^. The majority of patients with autoimmune lymphoproliferative syndrome (ALPS) carry heterozygous germline or somatic *FAS* mutations or both.

The role of FAS in maintaining lymphocyte homeostasis and peripheral immune tolerance to prevent autoimmunity was initially elucidated by studies of mice with deficient fas ** (MRL/ *lpr* knockout mice) or fasL (MRL/ *gld*) ^13, 14^. These mice were discovered to have massive DNT cell proliferation that accumulated in secondary lymphoid organs, in addition to hypergammaglobulinemia and glomerulonephritis ^13, 15^. Although *FAS* was identified as the critical gene underlying disease pathogenesis, the presence of the *FAS* mutation alone does not equal clinical disease manifestations. This was recently described in a comprehensive report by the National Institute of Allergy and Infectious Diseases (NIAID) of 150 ALPS-FAS patients and 63 healthy *FAS* mutation–positive family-member controls ^16^. While the predominant genetic mechanism of ALPS resides in the apoptosis-signaling complex by abnormal FAS proteins, healthy mutation-positive controls demonstrate apoptosis defects almost as severely as affected patients but can be clinically asymptomatic without elevated DNTs, sFASL, and IL-10. Moreover, some of the healthy mutation-positive controls had biomarker evidence of disease but were asymptomatic whereas other family members had very mild disease (for example, mildly low platelet count or very mild anemia). These data suggest that *FAS* mutations causing cellular apoptosis abnormalities alone are not sufficient to cause clinical ALPS. One hypothesis suggests that modifier genes as well as environmental factors may be involved ^17^. Furthermore, the clinical penetrance of heterozygous *FAS* mutations (described as 70% in the presence of one mutation) suggests that a “second hit” may be required for disease onset ^17, 18^. The precise mechanism underlying disease pathogenesis remains unclear but may be related to the development of DNTs, which are significantly elevated in patients with ALPS but lower in healthy controls.

Although the exact mechanism of *FAS* mutations leading to the accumulation of DNTs may not be clear, multiple studies demonstrate that DNTs are associated with a hyperactive mTOR pathway ^19, 20^. We initially hypothesized that hyperactive mTOR signaling may drive the abnormal proliferation of DNTs, based on our work in preclinical ALPS models demonstrating that the mTOR inhibitor sirolimus was effective in reducing DNTs, lymphadenopathy, splenomegaly, and autoantibodies in the *lpr* mice ^20^. We have since demonstrated the effective use of sirolimus in humans in a multi-institutional clinical trial of patients with ALPS refractory to standard therapy ^21^. Furthermore, DNTs were reduced in patients with ALPS yet normal T-cell subsets were relatively spared with sirolimus. Subsequently, Völkl *et al*. validated the critical role of dysregulated mTOR signaling demonstrating that DNT cells of patients with ALPS have enhanced mitotic activity and hyperactive mTOR signaling ^19^. Following treatment with sirolimus *in vitro*, DNT cells from these patients with ALPS demonstrate reduced proliferation and increased apoptosis. Furthermore, mTOR inhibition abolished expression of IL-10 in ALPS DNTs, which not only blocks proliferation but also selectively induces apoptosis in abnormally differentiated DNT cells. In contrast, DNT cells from patients whose ALPS was treated with mycophenolate mofetil (MMF) (also known as CellCept) retained the abnormal differentiation and mitotic activity, corroborating that mTOR is a more relevant target and suggesting sirolimus as a potentially superior therapeutic option compared with MMF.

## Genetics

Although ALPS can be caused by single-gene mutations, it is a complex human disease with variable disease penetrance and severity. Over 90 unique mutations are registered in the database of ALPS mutations at the NIAID ^22^. One of the first well-characterized human genetic diseases of apoptosis, ALPS is most commonly associated with autosomal dominant transmission of heterozygous germline mutations in *FAS*, described in up to about 70% of genetically defined ALPS ** ^13, 23, 24^. The next most common mutation includes somatic *FAS* mutations (10% of patients), or mutations that affect the FAS signaling apparatus known as the death-inducing signaling complex (DISC) ^16, 19, 25^. Mutations in genes encoding *FASL* and *CASP10* have been implicated in ALPS termed ALPS-FASL (<1%) and ALPS-CASP10 (<1%), respectively, but are much more rare ^16, 22^. As mentioned, some patients with ALPS have multiple mutations, including germline mutations in one *FAS* allele and somatic mutations in the other.

Older classification schemas designated the different genomic subtypes of ALPS with a numerical system (0–III) ( Table 2) ^2^. In 2009, a consensus conference at the National Institutes of Health (NIH) revised the nomenclature to mirror the World Health Organization system that uses a gene name–based classification for hematologic malignancies ^2^. The mode of inheritance for the majority of types is autosomal dominant (ALPS-FAS, ALPS-FASLG, and ALPS-CASP10). ALPS-FAS and ALPS-FASLG can also be caused by biallelic pathogenic variants inherited in an autosomal recessive manner ( Table 2). ALPS-sFAS and ALPS-FAS patients are clinically indistinguishable.

A number of ALPS-like syndromes with identifiable mutations not directly seen in ALPS are continually being discovered and described ^26^. Recent whole exome sequencing and whole genome sequencing have revealed new potential genetic drivers in the subgroup of ALPS with undetermined genetic defects (ALPS-U). Table 1 lists a number of candidate genes—including *KRAS*, *NRAS*, *CTLA4*, *LRBA*, *PI3kinase* (PI3κ), *MAGT1*, *STAT3*, and *TNFAIP3* (TNF-α–induced protein 3)—that have all been linked with ALPS-like features ^26– 32^. It is beyond the scope of this review to discuss all of these ALPS-like syndromes in detail. Table 1 provides key differences and considerations for diagnosis and management. A key point to emphasize is that these other syndromes are rare and, as is often the case in newly identified rare diseases, the published disease phenotype is based on a select number of patients who came to medical attention for a unifying clinical feature. As larger studies are performed, we may see that the published phenotype does not resemble the “actual” most common phenotype of the disease; for example, clinical outliers may represent the index cases. Furthermore, families with multiple mutations in ALPS and ALPS-like genes ( *CASP10* and *sFAS* ^33^ and *FAS*, *XIAP*, and *UNC13D* ^34^) have been described leading to complex phenotypes ^33, 34^. Interestingly, the genetic alterations in the Fas pathway influence not only the development of ALPS but also the phenotype—by the concurrent effect of other mutations. Therefore, it is important to have a low index of suspicion for ALPS and other ALPS-like disorders in children with unexplained lymphoproliferation or chronic autoimmune disease. Given the power of whole exome sequencing of DNA from patients with ALPS, we are likely to be at the precipice of having a better understanding of the pathogenesis of diseases with immune dysregulation and identifying a number of potential candidate genes that have features similar to those of ALPS.

## Clinical characteristics and epidemiology

In spite of the unifying gene defects found in ALPS, there is a wide range of disease symptoms and variability in the defining characteristics among individuals and subtypes of ALPS. The prevalence and true incidence of ALPS are unknown, likely since many instances remain undiagnosed or misdiagnosed. In fact, recent studies have shown that it may be more common than previously thought, given recognition of adult-onset disease and patients with a mild phenotype ^16^. Young patients with autoimmune manifestations early in life can be a diagnostic clue for underlying ALPS. Patients with ALPS can often manifest more severe disease characteristics in early childhood which resolve by adolescence and young adulthood ^35^. Thus, in retrospect, these patients with resolved clinical manifestations that were unexplained in the past likely carried the diagnosis of ALPS, leading to marked underestimation of true prevalence. There is also no known difference in severity of disease between sex, in spite of data suggesting a male preponderance. ALPS has been diagnosed in both sexes and in diverse racial backgrounds ^16^.

At some point in their lives, patients with ALPS, by definition, have clinically identifiable chronic lymphoproliferation, which manifests as adenopathy (>95% of patients) and splenomegaly (>90%) or hepatomegaly (40–50%) ^16, 36, 37^. The earliest and primary clinical manifestation of ALPS is chronic, diffuse lymphadenopathy, with or without splenomegaly or hepatomegaly or both, in an otherwise healthy child. The majority of patients develop lymphoproliferation at a young age (median age of 11.5 months), often in the absence of associated constitutional symptoms ^16^. The lymphoproliferation tends to improve with age and can wax and wane randomly but commonly worsens in adolescence before resolving in most patients in their early 20s. More than 80% of patients with ALPS experience a prolonged period of enlarged palpable and non-tender lymph nodes.

Patients with ALPS can have lymphocytosis that affects T and B cells but not natural killer cells. Although absolute numbers of total T and B cells are increased, the pronounced lymphoproliferation has been attributed mostly to the accumulation of the pathognomonic DNTs ^19^. The biologic hallmark of ALPS, DNTs are mature post-thymic T cells that express a rearranged CD3/TCRα/β receptor but lack CD4 or CD8 co-receptors ^5^. These cells are distinct from TCRγ/δ, which are normally CD4 ^−^CD8 ^−^, and can be increased non-specifically in ALPS and other conditions ^38^. In healthy adults, the DNT cells constitute less than 1% of peripheral lymphocytes, whereas in ALPS, DNTs can comprise more than 40% of lymphocytes ^38^. In addition to ALPS, DNTs have been found in the peripheral blood of patients with other autoimmune diseases like systemic lupus erythematosus, mixed connective tissue disease, and antinuclear antibody–positive oligoarticular or polyarticular juvenile idiopathic arthritis; however, their precise function or pathogenic role is not clear ^5^. In ALPS, in contrast to these other autoimmune diseases, the elevations of DNT cells are above 3% of total lymphocytes (or more than 5% of T lymphocyte cells) and this is rarely seen in conditions other than ALPS ^2, 39^. They have long been thought to arise from chronically active or senescent CD8 ^+^ T cells unable to undergo apoptosis because of defective Fas signaling. Recent studies have since challenged that notion with evidence of Ki-67 staining by immunohistochemistry of lymph nodes of patients with ALPS, demonstrating a unique phenotype with expression of both memory and markers of senescence, which also exhibit hyperactive mTOR signaling ^19^.

Autoimmunity is the second most common clinical manifestation, affecting over 70% of patients ^1, 13, 40^. The most common autoimmune manifestation is autoimmune destruction of blood cells in one or more cell lineages (that is, autoimmune hemolytic anemia, autoimmune thrombocytopenia, or neutropenia or a combination of these). This finding can be associated with either an elevated or decreased serum IgG. Although the presence of the cytopenias may not be present at the time of initial diagnosis, the evidence of autoimmune cytopenias—including Coombs-positive autoimmune hemolytic anemia and autoimmune thrombocytopenia with or without the association of autoantibodies—can be detected before manifestations of the autoimmune disease clinically ^35^. Autoimmune cytopenias may vary from asymptomatic laboratory abnormalities to multi-lineage cytopenia-related life-threatening illness ^40^. In many patients, the autoimmune cytopenias often require medical intervention—ranging from treatment for periodic disease flares after infections to chronic therapy. Autoimmune cytopenias can also fluctuate in severity and type over time (for example, affecting one cell line and changing to another with age). Similar to lymphoproliferation, autoimmune cytopenias can improve with age, although they are less likely to resolve completely in adulthood ^1^.

Other autoimmune disease manifestations outside of cytopenias can also be seen in patients with ALPS. These can occur in approximately 10–20% of patients with ALPS and can affect nearly any organ system. The most common are skin rashes (typically but not exclusively urticarial). Other autoimmune manifestations include immune-mediated pulmonary fibrosis, autoimmune thyroiditis, uveitis, Guillain-Barré syndrome, hepatitis, nephritis, gastritis, pancreatitis, colitis, transverse myelitis, cerebellar ataxia, myocarditis, and arthritis ^37^. Of note, many of these co-morbid autoimmune manifestations are most commonly described in patients with ALPS-U. Some of these patients with ALPS-U do not have ALPS but have an ALPS-like disorder as previously mentioned ( Table 1). For example, at our institution, a number of patients who met diagnostic criteria for ALPS-U were later identified to have mutations in *LRBA*, *CTLA4*, and *PI _3_Kδ* (Teachey *et al*., unpublished data).

Patients with ALPS also have an increased risk of secondary malignancies, most commonly both Hodgkin and non-Hodgkin lymphoma ^41^. This risk is estimated to be up to 60 to 150 times that of the general population and is most prevalent in *FAS* mutant ALPS ^16, 41^. Interestingly, the increased risk of lymphoma was once consistently documented only in *FAS* mutant ALPS and even higher among patients with dominant-interfering mutations in *FAS* but lower in patients with *FAS* haploinsufficiency ^37, 42^. Recently, a family with homozygous *FASLG* mutation was reported, presenting the first documented case of peripheral T-cell lymphoma ^43^. Together, these data suggest that there may be a correlation between the degree of disruption of the apoptotic signaling complex and the propensity to develop lymphoma ^16, 43^. Healthy family members with *FAS* mutations but no clinical evidence of ALPS are at increased risk for lymphomagenesis, underscoring the role of *FAS* as a tumor-suppressor gene. Co-morbid factors that have also been proposed to increase this risk include defective T-cell surveillance, Epstein-Barr virus (EBV) infection, and defective B-cell apoptosis ^16, 44^. However, general consensus concludes that abnormal immune regulation and defective FAS-mediated apoptosis provide an expanded lymphoid pool at risk for clonal transformation ^44^. The temporal delay (cumulative incidence increases with age) between the onset of ALPS manifestations and that of lymphoma also suggests a requirement for additional oncogenic events such as mutations in *C-MYC*, *CCND1*, *BCL2*, and *BCL6* ^45^. In fact, increased somatic hypermutation has been noted in cells with FAS-induced apoptotic defects, leading to self-reactive specificities, and also in dysfunctional immunoglobulins or in double-strand breaks that cannot be repaired by the normal DNA repair machinery.

## Differential diagnosis: whom to test?

No specific laboratory abnormality alone is diagnostic of ALPS. Given its heterogeneous phenotype, the constellation of lymphadenopathy, organomegaly, and autoimmunity can be found in other malignant, infectious, autoimmune, and rheumatologic conditions. For example, primary immunodeficiencies with autoimmune and other cytopenias, namely in the context of immune dysregulation, can have similar, if not overlapping, characteristics ^30^. Other lymphoproliferative disorders—such as Castleman disease, Rosai-Dorfman disease, X-linked lymphoproliferative disease, Dianzani autoimmune lymphoproliferative disease and Kikuchi-Fujimoto disease—can have clinical features similar to those of ALPS ( Table 1) ^4^. RALD with somatic pathogenic variants of *NRAS* and *KRAS*, CEDS, FADD deficiency, *PI3κ*, *LRBA*, *CTLA4*, GOF germline *STAT3* mutations, and common variable immunodeficiency 9 ( *PRKCD* deficiency) are considered ALPS-like disorders since they cause similar phenotypes, but the pathogenic gene variants may not be within the FAS/FASL pathway. Identification of the potential underlying mutation, as in GOF *STAT3* mutations, not only expands the clinical spectrum of ALPS-like disorders but also provides the rationale for the use of inhibitors of the pathway when the underlying defect has been identified ^31, 46, 47^.

Overlapping syndromes have also been reported among combined variable immunodeficiency (CVID), Evans syndrome (ES), and ALPS. In fact, a subset of patients with ALPS have co-morbid CVID; therefore, distinguishing between these two diseases can be difficult ^48, 49^. Autoimmune hematological abnormalities, specifically cytopenias, are the most common of all autoimmune manifestations of CVID ^50^. While most patients with ALPS have elevated IgG, they can also have decreased IgG levels, more characteristic of CVID. Similarly, ES, defined by autoimmune destruction of at least two hematologic cell types, can have a constellation of overlapping clinical findings, as in ALPS, and has been reported to precede the clinical and immunological phenotype of CVID ^49, 51, 52^. We have previously described that over one third of patients with diagnosed ES actually fulfilled the criteria and diagnosis of ALPS ^52, 53^. The underlying pathophysiology of ES is unknown but is thought to be secondary to generalized immune dysregulation. Therefore, ES is a diagnosis of exclusion, and other confounding disorders, like chronic EBV, must be ruled out before establishing the diagnosis ^43, 54^. Hyper IgM syndrome and *WAS*-related disorders that include Wiskott-Aldrich syndrome, X-linked thrombocytopenia, and X-linked congenital neutropenia can also have features similar to those of ALPS.

Certainly, continued discovery of genetic mutations in the subgroup of patients with ALPS-U will likely reveal other potential overlapping syndromes to ALPS. Although some of the criteria for ALPS are defined, further study is clearly needed.

## Diagnosis

Given the heterogeneity in clinical features, the diagnosis of ALPS is based on clinical observation and laboratory abnormalities, including elevated DNT cells in peripheral blood. Significant advances in our understanding of the disease since its initial identification have prompted revision of diagnostic criteria ( Table 3) and classification defined currently by the NIH consensus statement in 2009 ^2, 12, 13^.

**Table 3.** Current 2010 clinical criteria for the diagnosis of autoimmune lymphoproliferative syndrome.

Required criteria
Chronic (>6 months), non-malignant, non-infectious lymphadenopathy or splenomegaly or both
Elevated CD3 ^+^TCRab ^+^CD4 ^−^CD8 ^−^ “double negative T cells” (DNT) cells > 1.5% of total lymphocytes or 2.5% of CD3 ^+^ lymphocytes with normal or elevated lymphocyte counts
Accessory: primary
Defective lymphocyte apoptosis
Somatic or germline mutation in FAS, FAS ligand gene (FASLG), or caspase-10 gene (CASP10)
Accessory: secondary
Elevated plasma soluble FAS ligand (sFASL) (>200 pg/mL) or elevated plasma interleukin-10 (IL-10) levels or elevated serum or plasma vitamin B _12_ levels or elevated plasma IL-18 levels
Typical immunohistological findings as reviewed by an experienced hematopathologist
Autoimmune cytopenias and elevated IgG levels
Family history of a non-malignant/non-infectious lymphoproliferation with or without autoimmunity

Table adapted from Oliveira *et al*. ^2^. A definitive diagnosis of autoimmune lymphoproliferative syndrome (ALPS) comprises the required criteria plus one primary accessory criterion. Probable diagnosis of ALPS includes the required criteria with one secondary accessory.

The three classic diagnostic signs of ALPS are chronic lymphadenopathy and splenomegaly, a functional defect in lymphocyte apoptosis, and an increase in DNT cells ^2^. Based on the consensus statement, for the diagnosis of ALPS to be established, a patient has to meet both required criteria and one primary accessory criterion ( Table 3). Lymphoproliferation must be chronic (>6 months) and affect two distinct nodal regions (with or without splenomegaly) while excluding neoplastic and infectious etiologies. The pathognomonic DNTs must be elevated, exceeding 1.5% of total lymphocytes or 2.5% of T lymphocytes. Importantly, this criterion is specific to normal or elevated lymphocyte counts since the relative distribution of DNT cells in lymphopenia is not accurately known and may be falsely elevated ^2, 13, 26^. Additional accessory criteria are further divided into primary and secondary categories. Presumably to encompass a larger number of similar novel immune disorders that mimic ALPS, the revised diagnostic criteria distinguish probable from definitive ALPS diagnosis, including one secondary accessory criterion rather than one primary accessory criterion (as in definitive ALPS). Some patients with probable ALPS have an ALPS-like disorder and should be tested for these conditions. If these other disorders have been ruled out, then patients with probable ALPS should be treated similarly to patients with definitive ALPS. As new ALPS-like syndromes are identified, it is important to consider and evaluate for these conditions among those with probable ALPS or ALPS-U or both.

In a recent study following individuals with ALPS over 20 years compared with healthy mutation-positive relatives, Price *et al*. suggest that a novel biomarker signature, including elevated levels of soluble IL-10, IL-18, sFASL, and vitamin B _12_ measured in plasma/serum, can make a presumptive diagnosis of ALPS ^16^. Although this profile may not substitute for molecular analyses, since other conditions like CVID or ES can have a similar profile (namely elevated vitamin B _12_ and sFASL), this is an inexpensive set of tests that can prompt further testing, especially when molecular or genetic analysis may not be readily available. However, new insights and broader understanding of ALPS and ALPS-U highlight the need for continued revision of current guidelines to facilitate prompt diagnosis while recognizing the evolving complexity of the ALPS diagnosis arrived at through many potential pathways. Moreover, the overlapping clinical features and newly identified novel immunodysregulatory syndromes that may underlie overlapping symptoms suggest the need for prompt consideration by clinicians and potentially more advanced diagnostic tools, including both traditional assays or more modern techniques, such as next-generation sequencing or whole exome sequencing.

## Management

The management of ALPS focuses on treatment of disease manifestations and complications. The only known cure is HSCT. However, because of the risks associated with HSCT, it is often performed only in those with very severe clinical phenotypes who are refractory to immune suppression ^37^. The majority of patients with ALPS can live relatively normal healthy lives without the need for HSCT. Therefore, the bulk of management focuses on monitoring for and treatment of disease-specific complications, including lymphoproliferation and autoimmune cytopenias. The overall prognosis for patients with ALPS is good but depends on steroid-sparing management of cytopenias. Nearly 50–60% of patients with ALPS require immunosuppressive therapy to control autoimmunity, but the overwhelming majority of these patients can be managed with single-agent immune suppression ^37^. A careful risk-benefit evaluation is needed prior to exposing any patient to medications with long-term co-morbidities.

First-line treatment of ALPS often includes high-dose intravenous corticosteroids and intravenous immunoglobulin (IVIgG) ^13, 26^. Many patients often respond well to corticosteroids (1–2 mg/kg). Although in the short term the toxicities of high-dose steroids can be mild (including hypertension, hyperglycemia, irritability, and weight gain), the long-term toxicities are serious and potentially severe ^40^. Furthermore, since ALPS is a chronic disease, many patients need chronic treatment ^13, 40^. As a result, at our center, we try to limit corticosteroid exposure, with a low threshold to transition quickly to steroid-sparing immune suppression.

IVIgG, in contrast, is less effective, and it has a poorer response among patients except those with single-lineage autoimmune thrombocytopenia ^1, 6, 40^. Similar to steroids, IVIgG can be quick-acting (within 48 hours) and well tolerated; however, the effect is short-lived, requiring repeated infusions. Anti-D immunoglobulin (WinRho) is often discouraged for isolated thrombocytopenia since many patients may be positive for the direct antiglobulin test with a risk for worse hemolysis with additional WinRho administration ^1, 40^. Isolated neutropenia, either acute or chronic and while not otherwise being managed on maintenance therapy, may benefit from granulocyte colony-stimulating factor. However, we would recommend treating these patients with autoimmune neutropenia only if they develop infections.

Management strategies such as splenectomy and rituximab are commonly employed for autoimmune disease but are relatively contraindicated in patients with ALPS, especially in light of other more effective common therapies. Rituximab, a monoclonal antibody against the CD20 molecule, has been used but with variable efficacy ^48, 55^. Most available data regarding efficacy of rituximab arise from retrospective studies of patients with autoimmune hemolytic anemia and immune thrombocytopenia (ITP) ^55^. Unlike patients with ITP, some patients with ALPS treated with rituximab have been shown to never recover normal B-cell function after its use, resulting in persistent hypogammaglobulinemia. In the absence of prospective trials, the true benefit of rituximab is not known, and there is the added risk of requiring lifetime IVIgG replacement. Thus, we believe that rituximab should be avoided unless other treatment approaches fail.

In the past, ALPS has also been treated with splenectomy to manage chronic refractory cytopenias. A number of studies have demonstrated that patients with ALPS who undergo splenectomy have an increased risk of pneumococcal sepsis despite vaccination and antimicrobial prophylaxis. Though considered in refractory cases who fail all available options, splenectomy has significant risks, is often ineffective, and rarely leads to permanent remissions ^16, 56^. In one report of patients with ALPS, 41% of splenectomized patients developed one or more episodes of sepsis, and there were six deaths due to overwhelming sepsis, which reinforces the recommendation to avoid splenectomy and consider second-line immunomodulatory agents to manage chronic cytopenias ^26^.

**Figure 2.**
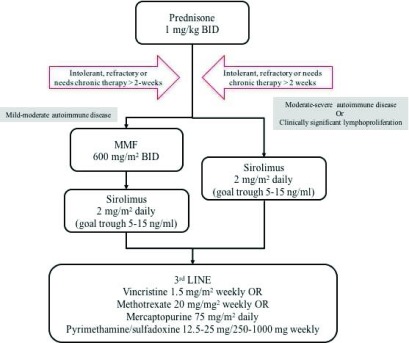
Proposed algorithm of our approach to the treatment of patients with autoimmune lymphoproliferative syndrome (ALPS) and associated mild-moderate and moderate-severe autoimmune disease, with or without clinically significant lymphoproliferation. Adapted from George *et al*. ^1^. BID, twice daily.

The two most commonly used immunomodulatory drugs for ALPS are MMF and sirolimus. MMF is metabolized in the body to mycophenolic acid (MPA) and inhibits inosine-5′-monophosphate dehydrogenase. MPA acts to cause a preferential reduction in guanosine nucleotides in T and B cells, thereby inhibiting proliferation ^57^. The administration of MMF, initially described in 2005, has proven to be an effective steroid-sparing agent in improving autoimmune cytopenias in approximately 80% of patients with ALPS, based on clinical trials ^40, 58^. Among its benefits are that MMF does not require therapeutic drug monitoring, does not have significant drug-drug interactions, and has a safe and tolerable side-effect profile. The most common side effects include diarrhea and neutropenia. In spite of measured improvements in autoimmune disease, MMF has not been observed to cause lymphocyte death or have any effect on lymphoproliferative disease or depletion of DNTs ^40, 59^. Moreover, some patients have partial responses, and in some patients the responses are not durable, requiring discontinuation of MMF or use of periodic steroid pulses during disease flares. As it is a well-tolerated agent that is effective in many patients, it is often used. Therefore, MMF is still recommended as a second-line agent, particularly in patients with mild to moderate autoimmune disease without clinically significant lymphoproliferation ( Figure 2). Of note, unlike corticosteroids, second-line therapies often take a few weeks and occasionally months to demonstrate benefit. Thus, patients often need to remain on steroids, which are slowly tapered. If tolerated, a 3- to 6-month trial should be performed before considering a second-line therapy a treatment failure.

Sirolimus is gaining increased recognition as an extremely effective agent in patients with ALPS. The primary rationale for using sirolimus stemmed from preclinical data from our group and others demonstrating dysregulation of mTOR signaling in patients with ALPS ( 6, 20; see pathogenesis). These data have been further validated by other studies demonstrating that the pathognomonic DNT cells, as well as a subset of CD4 and CD8 T cells in patients with ALPS, are highly proliferative *in vivo* associated with a hyperactive mTOR pathway ^19^. Other potential benefits of mTOR inhibition are evidence of promotion and expansion of FoxP3 ^+^ regulatory T cells (Tregs) both *in vitro* and *in vivo*, often implicated in autoimmune disease pathogenesis ^60, 61^.

We initially published the results using sirolimus with ALPS patients in a small retrospective cohort ^59^. Based on our preclinical data, the compelling rationale, and the results in our retrospective cohort, we opened a multi-center prospective clinical trial of sirolimus in patients with ALPS as well as children without ALPS and chronic autoimmune cytopenias. We demonstrated successful use of sirolimus in children with refractory autoimmune multi-lineage cytopenias, and 100% of patients with ALPS demonstrated a complete response (CR) or near CR. These included rapid durable improvements in autoimmune disease, lymphadenopathy, and splenomegaly within 1–3 months of starting sirolimus that were also durable, and follow-up extended up to 10 years ^21^. In addition to the ALPS patients treated in this study, we have also guided clinicians to treat 35 additional ALPS patients with sirolimus. These patients were diagnosed and treated at our institution, other centers in the United States, and 18 different countries, including nations on six continents ( Table 4). As these patients were not enrolled in our trial, we cannot present definitive data, but reportedly the majority (31 out of 35) of those patients achieved a durable CR with sirolimus.

**Table 4.** An additional 35 patients from 18 different countries successfully used sirolimus for patients with autoimmune lymphoproliferative syndrome but were not enrolled on the study.

North America	USA
	Canada
South America	Brazil
Europe	United Kingdom
	France
	Germany
	Italy
	Kazakhstan
	Poland
	Spain
	Sweden
Africa	Egypt
	South Africa
Asia	China
	India
	Russia
Middle East	Saudi Arabia

Additional published studies have since demonstrated similar results, suggesting moving sirolimus to upfront, first-line therapy ^7, 19, 62^. In a recent retrospective study of 18 patients with definitive ALPS (9 type 1a, eight sFAS, and one homozygous) treated with sirolimus as first-line therapy, the majority (94%, 17 out of 18 patients) experienced a CR. In addition, those with a CR had an associated normalization of known biomarkers, including DNTs and serum levels of vitamin B _12_, IL-10, and sFASL ^7^. MMF has similarly been shown to be effective in improving autoimmune cytopenias; however, unlike with sirolimus, many of these patients have partial responses prompting continued steroid use while others have relapsed ^58, 63^. Although some providers believe MMF should be trialed before sirolimus, Völkl *et al*. demonstrate that DNT cells treated with MMF retain abnormal differentiation and mitotic activity, indicating that sirolimus may be superior to MMF therapy ^19^. Furthermore, those patients who previously fail MMF often respond to sirolimus ^63^. It is uncertain whether sirolimus affects expansion or induces apoptosis of DNT cells or both, but its therapeutic efficacy suggests specific signaling requirements of mTOR in DNT cells. Furthermore, as previously mentioned, various studies have suggested that sirolimus promotes generation, expansion, and functionality of Tregs ^61^. However, the role of sirolimus in its contribution to modulating Tregs and their role in self-tolerance remain unclear.

As described by our multi-institutional prospective trial, we do not see any deficits in immune function in children with ALPS being treated with sirolimus ^21^. Importantly, none of those children was functionally immunosuppressed, as demonstrated by intact, unchanged functional immune activity in spite of prolonged treatment with sirolimus, without an increased incidence of opportunistic infection. Therefore, we do not recommend pneumocystis, antifungal, or antibacterial prophylaxis in ALPS patients on single-agent sirolimus. In contrast, we found that some patients with ALPS treated with MMF become profoundly lymphopenic.

One of the primary drawbacks of sirolimus is the need to monitor serum levels. We typically target our dosing to achieve a trough of 5–15 ng/mL, which often approximates to a dose of 2.5 mg/m ^2^ daily. Short-term side effects include mucositis, hypertension, and hypertriglyceridemia. We have not witnessed a correlation between targeted trough values and the presence of side effects; however, a dose-toxicity relationship has been shown in larger trials using sirolimus after solid organ transplant. In fact, we would like to highlight that most of the published data using sirolimus and other mTOR inhibitors are derived from combination therapies for graft rejection after transplant, or in malignancy, and only a handful of studies were directed to monotherapy. Other known issues with mTOR inhibitors include monitoring for hyperlidemia, decreased renal function, and myelosuppression. Oral mucositis can occur and is most pronounced in the first month of therapy and often resolves with time. Additionally, sirolimus pharmacokinetics, unlike MMF, can be altered by other multiple medications. Therefore, it is crucial to check for drug-drug interactions anytime a new medication is added. Patients who develop hyperlipidemia may need medical therapy with fish oil or a statin.

Our current proposed outline for our approach to patients with ALPS is shown in Figure 2. We continue to recommend MMF in patients with mild to moderate autoimmune cytopenias that do not have clinically significant lymphoproliferation (organ compromise or hypersplenism). However, for patients who fail MMF, have moderate to severe autoimmune cytopenias, or have clinically significant lymphoproliferation, we recommend sirolimus. More and more frequently, our group and others are using sirolimus as first-line therapy. Third-line agents and their dosing are also described in Figure 2.

More evidence is emerging supporting the use of sirolimus given its safety, tolerability, and most importantly efficacy. This is especially true in regard to ALPS-FAS patients and therefore may not be applicable to all patients. Certainly, the discovery of other pathogenic mechanisms of diseases like ALPS and other primary immunodeficiency and autoinflammatory disorders will likely reveal other specific immunomodulators that can target a particular arm or cytokine of the immune system, such as abatacept for *CTLA4 deficiency* (CHAI or LATAIE), tocilizumab in GOF STAT3 defects, or ruxolitinib for GOF STAT1 defects ^48^ ( Table 1). Patients with PI _3_KCD GOF mutations have also been shown to have hyperactive mTOR activity, which has since prompted the use of sirolimus successfully for these patients ^29, 48^. Therefore, the new insights and increased attention of these diseases not only highlight the evolving complexity of ALPS and ALPS-like disorders but also emphasize the need for a broader understanding of the immune dysregulation underlining ALPS and ALPS-like disorders.

Beyond mTOR inhibitors and MMF, other agents trialed in patients with ALPS have been met with variable success. Pentostatin has been reported to have limited efficacy in some children with refractory cytopenias ^13, 26, 48, 56, 58, 64^. Pentostatin is an irreversible inhibitor of adenosine deaminase (ADA), which leads to the accumulation of intracellular deoxyadenosine-5′-triphosphate (d-ATP) that results in cytotoxicity and cell death by activation of apoptosis. Its use has been demonstrated to lead to improvement in hyperleukocytosis and decrease in the frequency of red cell and platelet transfusion. We have also successfully used a combination of methotrexate and sirolimus in ALPS-like patients who fail monotherapy sirolimus. Alternatively, we have treated highly refractory patients with autoimmune cytopenias with acute lymphoblastic leukemia maintenance-like therapy, including mercaptopurine and oral methotrexate with vincristine/prednisone pulses, for those experiencing flares. Finally, we have successfully treated some ALPS-like patients with the proteasome inhibitor bortezomib. Rarely, HSCT has been used for refractory patients. The experience overall is limited and careful consideration is needed given the significant risks associated with HSCT.

## Surveillance

Whereas non-malignant lymphoproliferative manifestations often regress or improve over time, the risk for development of lymphoma is lifelong. As previously mentioned, the risk of an ALPS patient developing Hodgkin lymphoma is estimated at 150 times that of the general population, and the risk of non-Hodgkin lymphoma is increased by 14-fold in those patients ^4, 41^. Given the fluctuation in size of chronic generalized adenopathy in patients with ALPS, these patients need close clinical observation; however, no imaging modality, including 18-fluorodeoxyglucose-positron emission tomography (FDG-PET), can accurately distinguish benign from malignant lymphoproliferation as ALPS lymphadenopathy is PET-avid ^13^. Furthermore, early diagnosis of lymphoma does not change clinical outcome. Therefore, we opt to evaluate for malignancy only in patients who have a significant change in disease pattern or who develop constitutional symptoms. Since progression to lymphoma is associated with monoclonal expansion of malignant lymphocytes, occasionally testing for clonality in lymphocyte subsets can help distinguish benign from malignant disease.

## Summary

Early recognition and diagnosis of ALPS are integral to successful management and treatment. Generally, the prognosis is good, especially in light of improved steroid-sparing agents, including sirolimus. Given robust preclinical and clinical evidence, we believe sirolimus should be considered as first-line therapy in many patients with ALPS, as it is an effective targeted therapy for the disease.
